# Effects of Microtopography on Neighborhood Diversity and Competition in Subtropical Forests

**DOI:** 10.3390/plants14060870

**Published:** 2025-03-11

**Authors:** Jianing Xu, Haonan Zhang, Yajun Qiao, Huanhuan Yuan, Wanggu Xu, Xin Xia

**Affiliations:** Innovative Research Team for Forest Restoration Mechanisms, Chishui National Ecological Quality Comprehensive Monitoring Stations, Research Laboratory for Protected Area Survey and Monitoring, Nanjing Institute of Environmental Sciences, Ministry of Ecology and Environment (MEE), Nanjing 210042, China

**Keywords:** neighbor scale, neighborhood diversity, competition index, microtopographic, subtropical forest

## Abstract

Forests are complex systems in which subtle variations in terrain can reveal much about plant community structure and interspecific interactions. Despite a wealth of studies focusing on broad-scale environmental gradients, the role of fine-scale topographic nuances often remains underappreciated, particularly in subtropical settings. In our study, we explore how minute differences in microtopography—encompassing local elevation, slope, aspect, terrain position index (TPI), terrain ruggedness index (TRI), and flow direction—affect neighborhood-scale interactions among plants. We established an 11.56-hectare dynamic plot in a subtropical forest at the northern margin of China’s subtropical zone, where both microtopographic factors and neighborhood indices (density, competition, diversity) were systematically measured using 5 m × 5 m quadrats. Parameter estimation and mixed-effects models were employed to examine how microtopography influences plant spatial patterns, growth, and competitive dynamics across various life stages. Our findings demonstrate that aspect and TPI act as key drivers, redistributing light and moisture to shape conspecific clustering, heterospecific competition, and tree growth. Remarkably, sun-facing slopes promoted sapling aggregation yet intensified competitive interactions, while shaded slopes maintained stable moisture conditions that benefited mature tree survival. Moreover, in contrast to broader-scale observations, fine-scale TRI was associated with reduced species richness, highlighting scale-dependent heterogeneity effects. The intensification of plant responses with life stage indicates shifting resource demands, where light is critical during early growth, and water becomes increasingly important for later survival. This study thus advances our multiscale understanding of forest dynamics and underscores the need to integrate fine-scale abiotic and biotic interactions into conservation strategies under global change conditions.

## 1. Introduction

Forest ecosystems, as the dominant terrestrial systems, play an indispensable role in global carbon and water cycles as well as in the maintenance of biodiversity. The structure, composition, and functionality of forests are not static; even within landscapes that appear homogeneous at broader scales, there exists substantial fine-scale spatial variation [1,2,3,4,5,6,7]. This fine-scale heterogeneity is expressed not only in the spatial patterns of species composition and diversity but also in the complex neighborhood interactions among individual plants, and the degree of variation at this scale often rivals that observed across broader environmental gradients or biogeographic regions [8,9]. At even smaller scales, the underlying complexity may be determined by a combined influence of abiotic factors—such as microtopography, soil ruggedness, and moisture gradients—and species interactions. These interdependent factors influence both the vertical and horizontal structure of forests, resulting in canopy differentiation, understory diversity, and overall ecosystem productivity [1,2,5]. Therefore, a deep understanding of this heterogeneity and its effects on the mechanisms governing plant competition and diversity not only provides insight into how secondary forests adapt and recover under global change but also helps to identify the key processes that maintain ecosystem functionality and resilience, which is crucial for devising effective forest conservation and management strategies.

In the formation of forest heterogeneity, the interactions among neighboring plants are pivotal ecological processes that determine community structure and dynamics [10,11,12]. Under limited resource conditions, plant individuals compete for access to light, water, and nutrients; this competition occurs both interspecifically and intraspecifically [5,10,11,12,13,14]. The observed changes in forest communities across different topographic settings are directly related to variations in soil resources (e.g., water and nutrients), which in turn modify plant–plant interactions [15,16,17]. At the neighborhood scale, the intensity and outcome of competition are affected by numerous factors, including intrinsic plant traits (e.g., growth rate, reproductive strategy, and resource use efficiency), as well as environmental conditions (e.g., light availability, water supply, and soil nutrients) [7,18,19]. The neighborhood competition index (NCI) is a critical metric for quantifying the intensity of competition among individual plants; it incorporates factors such as the size, distance, number, and species identity of neighboring plants, thereby providing a comprehensive measure of the competitive pressure experienced by a target individual [10,11,12,20]. NCI can be further decomposed into the conspecific NCI (CNCI) and the heterospecific NCI (HNCI), which respectively reflect the competitive interactions between a target plant and its conspecific and heterospecific neighbors [10,11]. Additionally, the neighborhood species richness (NSR) index, which quantifies the number of species present within a specified vicinity of a target plant, serves as an indicator of local community diversity [20,21,22]. Consequently, quantifying the relationships between neighborhood indices—such as plant spatial distribution, growth status, NCI, and NSR—and the spatial variation of environmental factors at corresponding scales is an essential approach for understanding spatial heterogeneity and forest diversity across multiple scales.

Topography is one of the key factors influencing community heterogeneity and neighborhood interactions among plants [4,5]. From large to small scales, topographic features shape forest structure and composition by influencing environmental factors. At global and regional scales, elevational gradients primarily drive variations in climatic factors such as temperature and precipitation, which in turn affect the composition of plant communities and the distribution of functional types [4,8,9]. In addition, regional topographic patterns influence atmospheric conditions—such as humidity and wind direction—thus creating diverse vegetation types and community characteristics at the landscape scale [4]. At the local scale, variations in mountainous terrain exert a major influence on plant distribution [7,18,19]. For example, areas such as ridges and steep slopes typically have shallow, well-drained soils that result in reduced water and nutrient availability, favoring species that are drought- and nutrient-stress tolerant [18,19,23,24]. In contrast, valleys and shady slopes often possess deeper and more fertile soils, along with better water conditions [7,23,25,26,27].

At even smaller scales, microtopography, as a fine-scale expression of topographic heterogeneity at the neighborhood scale [5,14,27], induces spatial variation at the meter level (e.g., small mounds, depressions, and shallow gullies) that leads to an uneven distribution of resources such as light, water, and nutrients. These spatial differences may create microclimatic variability [5,27,28], which in turn influences soil formation, water infiltration, and forest regeneration dynamics, thereby directly affecting the distribution, growth, and competitive interactions among individual plants. This influence is manifested in two main ways. First, by altering microhabitat conditions, microtopographic variability affects the point pattern distribution and growth of plant individuals. For example, spatial variability in microtopography can lead to differences in aspect or slope position, thereby indirectly influencing the light conditions received by individual plants, which affects photosynthetic performance and growth [5,20,29]. Seed dispersal and establishment are also susceptible to microtopographic effects, as seeds may tend to accumulate in depressions, thereby influencing the spatial distribution and competitive dynamics among plants [5,12,13,14]. Moreover, microtopography can affect litter accumulation and decomposition, thereby influencing nutrient cycling and plant growth. Second, by modifying the availability of resources, microtopographic variation indirectly alters neighborhood competitive interactions and diversity patterns [29,30]. For example, while sun-facing slopes and high-elevation areas provide greater light availability, they also incur more intense evapotranspiration, which impedes soil moisture and nutrient retention. This environmental scenario may facilitate the natural regeneration of heliophyte species; however, it also leads to stronger competition and higher mortality rates [5,10,11,12,23]. In summary, the heterogeneity of microenvironments at the neighborhood scale can redistribute resources such as light and water by altering their micro-scale availability, including changes in soil moisture, nutrient distribution, light intensity and direction, and temperature. These alterations have significant implications for plant competitive interactions and diversity patterns. Nonetheless, research on how microtopography influences the spatial distribution, growth, resource competition, and diversity of plants at the neighborhood scale remains relatively scarce, particularly in subtropical forest ecosystems, where the mechanisms by which microtopographic factors affect plant competitive intensity, modes of competition, and the resulting local diversity patterns require deeper exploration.

To address these gaps, we established an 11.56-hectare permanent monitoring plot (340 m × 340 m) within the Yaoluoping Nature Reserve, located at the northern margin of the subtropical zone in Anhui, China. This plot was subsequently subdivided into 4624 working quadrats (each 5 m × 5 m in size) to quantify fine-scale microtopographic variation at the neighborhood scale. In addition, detailed surveys were conducted within each quadrat, during which all woody tree species (with a DBH > 1 cm) were mapped, measured for DBH and height, and recorded by species identity and stem count. These data enabled us to quantify the spatial point pattern, growth status, and competitive relationships of individual plants at the neighborhood scale and to build statistical relationships between various microtopographic factors and neighborhood indices of growth, resource competition, and diversity through parameter estimation. This approach aims to transcend conventional landscape-scale analyses by incorporating fine-scale spatial heterogeneity, thereby elucidating the mechanisms by which microtopography influences plant growth, competition, and diversity assembly in subtropical forests and providing a theoretical framework for predicting community dynamics and informing conservation practices under global change.

## 2. Results

### 2.1. Effects of Microtopographic Factors on Plant Density, Size, and Competitive Interactions

**Figure 1:**
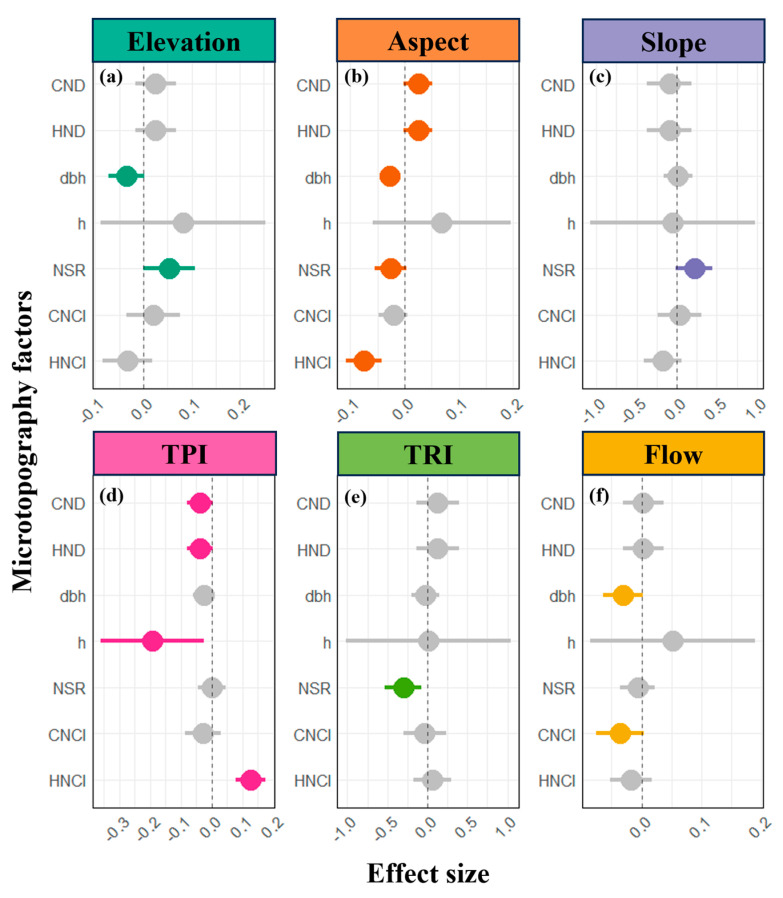
Parameter estimates for the effects of microtopographic factors (**a**–**f**) on seven neighborhood effect metrics at a scale of 5 m, with other test scales set at 2.5 m and 10 m; see Figure S1 in the Supplementary Files. Dots represent estimated parameter effects, with error bars indicating standard errors. A semi-transparent gray dashed line indicates a null effect (parameter estimate of zero) in each subplot.

In this study, we employed parameter estimation methods to assess the impacts of six microtopographic factors—elevation, aspect, slope, terrain position index (TPI), terrain ruggedness index (TRI), and flow direction index (flowdir)—on seven neighborhood effect metrics, i.e., conspecific neighborhood density (CND), heterospecific neighborhood density (HND), diameter at breast height (DBH), tree height (h), neighborhood species richness (NSR), conspecific neighborhood competition index (CNCI), and heterospecific neighborhood competition index (HNCI). The results revealed that aspect and TPI exerted the most pronounced influences on the various neighborhood metrics. Specifically, aspect exhibited a positive effect on both CND and HND (Figure 1b), suggesting that the environmental conditions on sun-facing slopes facilitate the clustering of individual plants. Simultaneously, aspect showed a marginal negative influence on NSR, potentially leading to a slight reduction in local diversity (Figure 1b). In contrast, changes in aspect implying a shift toward shaded slopes resulted in significantly negative effects on DBH, HNCI, and NSR, indicating that shading may constrain tree growth and distribution, while concurrently reducing resource competition among individuals—although the effect on conspecific competition was not statistically significant. Notably, the flow direction index produced influence patterns very similar to those observed for aspect: it negatively affected DBH, NSR, and both CNCI and HNCI (despite the non-significant effects on NSR and HNCI). This similarity suggests that variations in moisture distribution may serve as a key environmental factor limiting tree distribution, growth, and competition on shaded slopes.

The terrain position index (TPI) significantly and negatively impacted both conspecific and heterospecific neighborhood densities (CND and HND), as well as tree height (h) (Figure 1d). This finding indicates that, at fine scales, positions characterized by higher relative elevation, as opposed to lower depressions, constrain plant clustering and distribution, while also inhibiting vertical growth—a phenomenon likely associated with the spatial allocation of water resources under varying microtopographic conditions. Conversely, TPI demonstrated an extremely significant positive effect on the heterospecific neighborhood competition index (HNCI), suggesting that competition among plants intensifies at higher positions. This pattern may be related to the differential acquisition and allocation of light resources, with competition being more pronounced on sun-exposed slopes than on shaded types. Interestingly, our analysis of the terrain ruggedness index (TRI) revealed that, contrary to the commonly observed positive relationship between high variability and high diversity at broader scales, spatial variability at the neighborhood scale had a significantly negative effect on NSR (see Figure S1e,e1 in the Supplementary Files). In other words, an increase in terrain ruggedness at the fine scale appears to inhibit the enhancement of species diversity.

In summary, aspect and TPI emerged as the most influential microtopographic factors for determining neighborhood effects, primarily by modulating the distribution of light, moisture, and other resources that affect plant aggregation, growth, and competition. Elevation and TRI influence community structure through their contributions to landscape complexity and heterogeneity, while the marginally significant effects of flowdir and slope highlight the role of hydrological and topographic conditions in shaping plant growth and competition. Collectively, these findings underscore the multifaceted ways in which microtopography can significantly affect the spatial distribution and competitive interactions within plant communities.

### 2.2. Effects Across Life History Stages

Analyses across different life history stages reveal that the responses of plant density, size, and competitive interactions to microtopographic factors become increasingly pronounced as individuals progress from early to later developmental stages. Specifically, our results indicate that mature trees (adults) tend to exhibit stronger relationships—or trends that approach significance—with specific microtopographic variables, reflecting a stage-dependent habitat preference.

**Figure 2:**
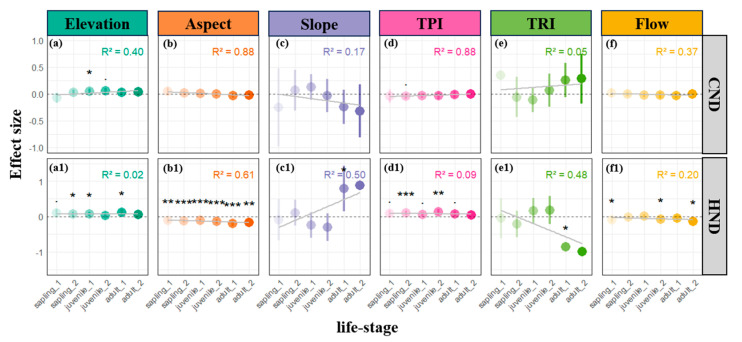
Parameter estimates for the effects of microtopographic factors on conspecific neighborhood density (CND: **a**–**f**) and heterospecific neighborhood density (HND: **a1**–**f1**) across life stages at a scale of 5 m. Dots represent estimated coefficients, with error bars depicting standard errors. Positive coefficients indicate positive effects, while negative coefficients indicate negative effects. Significance levels: • *p* < 0.1; * *p* < 0.05; ** *p* < 0.01; *** *p* < 0.001. In the inset figure, R-squared values represent the regression coefficient for changes across life stages, and light gray lines illustrate the trend of effects across life stages. A semi-transparent gray dashed line indicates a null effect (parameter estimate of zero) in each subplot.

In our analysis of plant population density, we observed that the effects of aspect and TRI on both conspecific and heterospecific densities became more distinct with advancing life stages (Figure 2b,b1,e,e1). Although some of these effects did not reach statistical significance in every stage, the overall trend suggests that as plants grow, they increasingly exhibit preferential associations with particular topographic conditions. Notably, the influences of slope and TPI on conspecific and heterospecific densities displayed completely opposite trajectories across successional stages. Specifically, the initially positive effect of slope on conspecific neighborhood density (CND) gradually diminished and eventually inverted to a negative impact, while its negative effect on heterospecific neighborhood density (HND) likewise weakened and reversed to a positive trend. Concurrently, the TRI’s positive effect on CND progressively intensified, whereas its influence on HND shifted from positive to negative (Figure 2e,e1). This pattern indicates that, as succession advances, increased microtopographic heterogeneity promotes conspecific clustering, while simultaneously suppressing the aggregation of heterospecific individuals.

**Figure 3:**
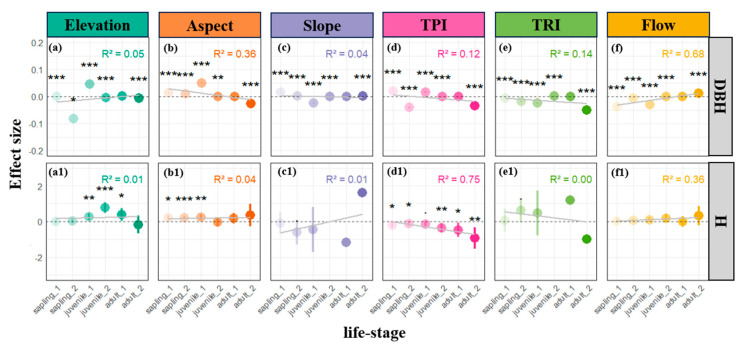
Parameter estimates for the effects of microtopographic factors on individual tree diameter at breast height (DBH: **a**–**f**) and height (H: **a1**–**f1**) across life stages at a scale of 5 m. Dots represent estimated coefficients, with error bars depicting standard errors. Positive coefficients indicate positive effects, while negative coefficients indicate negative effects. Significance levels: • *p* < 0.1; * *p* < 0.05; ** *p* < 0.01; *** *p* < 0.001. In the inset figure, R-squared values represent the regression coefficient for changes across life stages, and light gray lines illustrate the trend of effects across life stages. A semi-transparent gray dashed line indicates a null effect (parameter estimate of zero) in each subplot.

With respect to plant size, our results indicate that the effect of flow direction on both tree height and diameter at breast height (DBH) becomes increasingly pronounced over the life span of the plants (Figure 3f,f1). This trend implies that as trees grow—and their demand for water intensifies—the influence of moisture distribution becomes more critical, particularly for DBH development. In contrast, the negative effects of TPI on both tree height and DBH steadily strengthen as plants mature (Figure 3d,d1), with the impact on height being especially significant (R² = 0.75). This suggests that even subtle raised terrain features, such as minute ridges within the study area, can impose considerable constraints on vertical and radial growth, likely due to their reduced capacity for moisture retention. Such observations further substantiate the notion that increasing water demand during later developmental stages makes plants more sensitive to microtopographic influences.

**Figure 4:**
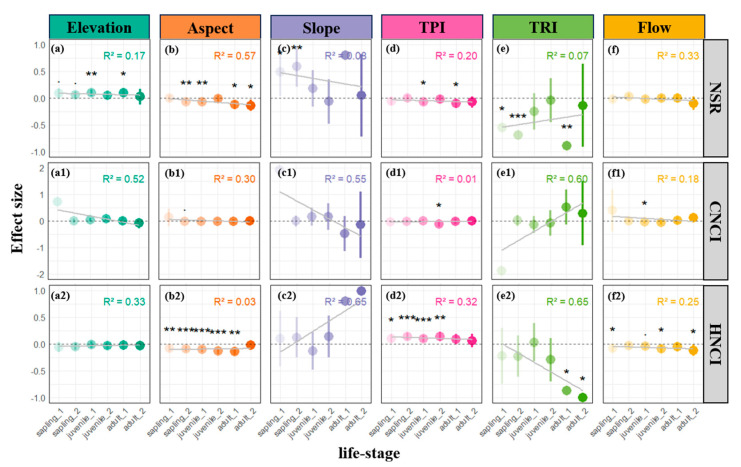
Parameter estimates for the effects of microtopographic factors on neighborhood species diversity (DBH: **a**–**f**), conspecific competition indices (H: **a1**–**f1**), and heterospecific competition indices (H: **a2**–**f2**) across life stages at a scale of 5 m. Dots represent estimated coefficients, with error bars depicting standard errors. Positive coefficients indicate positive effects, while negative coefficients indicate negative effects. Significance levels: • *p* < 0.1; * *p* < 0.05; ** *p* < 0.01; *** *p* < 0.001. In the inset figure, R-squared values represent the regression coefficient for changes across life stages, and light gray lines illustrate the trend of effects across life stages. A semi-transparent gray dashed line indicates a null effect (parameter estimate of zero) in each subplot.

Consistent with the trends observed in CND and HND, a similar trend with emerged in our assessment of neighborhood competition indices. For both the conspecific (CNCI) and heterospecific (HNCI) metrics, the negative impact of TRI on competition among neighboring individuals became modified with plant development: the effect on CNCI transitioned from negative to positive (albeit non-significantly), whereas the negative influence on HNCI became even more pronounced (Figure 4e1,e2). These findings suggest that greater terrain complexity may intensify intraspecific competition while mitigating interspecific competition, particularly among mature individuals in later successional stages.

## 3. Discussion

Microtopographic factors play a critical role in forest ecosystems by influencing species density, diversity, tree growth, and competitive interactions, which in turn shape forest structure and function. In this study, aspect and the terrain position index (TPI) emerged as the most significant microtopographic drivers of neighborhood effects; their influence likely operates through the spatial redistribution of essential resources such as light and water (Figure 1). Unlike traditional studies that have documented a relatively consistent positive relationship between high spatial heterogeneity and species diversity at broad scales, our findings reveal that the terrain ruggedness index (TRI) exerts a negative effect on neighborhood species richness (NSR) at the fine (5 m) scale, thereby enriching traditional habitat heterogeneity theory with insights derived from small-scale environments.

Specifically, the positive effect of aspect on neighborhood density (both CND and HND), coupled with its negative influence on resource competition indices (Figure 1d), indicates that small-scale variations in slope orientation significantly affect species distribution and competitive dynamics. In these patchy habitats, differences between slopes facing the sun and those facing away from it can lead to differential redistribution of light resources, which in turn, shapes plant distribution via two possible mechanisms. On one hand, sunlit patches receive more abundant light, which can provide a superior regeneration environment; indeed, several studies have reported the clumped regeneration of saplings under canopy gaps in tropical and subtropical secondary forests [5,23,30]. Our observation that sun-facing patches are predominantly occupied by saplings and juvenile trees (see Figure 2b) strongly supports this viewpoint. At the same time, enhanced evapotranspiration on sunlit slopes exacerbates water limitations, prompting conspecifics to adopt clumping strategies to reduce water competition [2,5,15,29]. On the other hand, although the shaded patches on (or facing) the opposite slope may limit tree growth and distribution to some extent (Figure 1b), they concurrently reduce the intensity of local resource competition. Even though the effect on conspecific resource competition is statistically insignificant, similar trends can be discerned (Figure 1b). This finding is consistent with the mode of light-mediated competitive interactions observed among tree saplings in subtropical forests, wherein the lower competitive intensity experienced by saplings on shaded slopes translates into higher survival rates [5]. Interestingly, the flow direction index (flowdir) produced effects very similar to those of aspect on DBH, NSR, and both conspecific and heterospecific neighborhood competition indices (CNCI and HNCI, respectively)—albeit with NSR and HNCI effects not reaching statistical significance—implying that moisture-related factors may be one of the primary abiotic constraints on tree distribution, growth, and competition on shaded slopes (Figure 1f). The positive effect of TPI on heterospecific competition (*p* < 0.001) reflects the role of elevated terrain (e.g., small mounds) and depressions (e.g., shallow gullies) in redistributing resources. Under gravitational influence, microtopographic features analogous to low, steep slopes along ridges poorly retain moisture and nutrients [28,31,32,33]. Consequently, higher TPI values may lead to the aggregation of soil moisture and nutrients in lower positions [15,31,32,33,34], thereby explaining our observation of a negative effect of TPI on neighborhood density and a more pronounced heterospecific resource competition at higher terrains (Figure 1d). In essence, plant individuals, both conspecific and heterospecific, tend to aggregate in depressional areas, whereas those on adjacent, slightly elevated slopes encounter more intense resource competition. Similar changes in species distribution and competitive relationships along topographic gradients have been reported in tropical forests, where higher nutrient and water availability in low-lying areas correlates with increased tree abundance and diversity, and saplings from depressions experience lower competitive pressure than those from higher locations [16,34,35,36,37].

Analyses across different life stages reveal that plants adopt stage-specific adaptive strategies and habitat-filtering effects in response to microtopographic variation (Figure 2, Figure 3 and Figure 4). As discussed previously, the overall pattern of conspecific and heterospecific aggregation observed in sunlit patches (Figure 1b) primarily stems from the aggregated distribution of saplings (Figure 2b,b1). Beyond the light-gap regeneration mechanism [5,23,29], the heightened conspecific aggregation (CND) observed during the sapling stage on sun-facing slopes may also be attributable to enhanced local water stress, which drives seedlings to aggregate as a means of ameliorating microenvironmental conditions [35,38]. Furthermore, as individual plants develop, we observed a gradual decline in conspecific competitive intensity on shaded slopes, while heterospecific resource competition consistently remains at a lower level (Figure 4b1,b2). The observation of higher NSR in shaded patches (Figure 4b), combined with the increased water demand associated with larger DBH and tree height at later life stages (Figure 4f,4f1), indicate that the stable moisture conditions on shaded slopes progressively become critical for mature tree survival—a pattern consonant with previous reports from warm temperate, subtropical, and tropical forests [5,28,38]. In essence, shaded slopes mitigate the increasing water demand imposed by growth (increased DBH and height) by reducing evaporation and maintaining soil moisture. This life-history-dependent habitat filtering suggests that microtopography regulates community assembly via a “stage-specific resource allocation” mechanism, with light resources dominating during the seedling stage and water and nutrient competition becoming more prominent during later stages [21,22,26]. In summary, the combined effects of aspect, TPI, and flow-related factors indicate that the light redistribution induced by microtopographic variability allows sun-facing ridges to allocate more abundant light, favoring the regeneration of saplings despite stronger resource competition; conversely, depressional areas on shaded slopes are more conducive to long-term survival. This implies that while sunlit slopes may offer superior light conditions for some species, the aggregated individuals also face substantial resource competition [18,19,22], whereas the relatively lower light, but improved moisture conditions, on shaded slopes favor individual survival and growth [21,22,26].

On the other hand, our study observed a significant inhibitory effect of the terrain ruggedness index (TRI) on neighborhood species richness (NSR), thereby enriching the habitat heterogeneity theory across spatial scales—from global to landscape levels [4]. Traditional research, which has largely focused on broader scales, typically documents a significant positive correlation between species diversity and topographic complexity (topographic heterogeneity, TH), whereby complex terrain promotes the coexistence of diverse species in heterogeneous environments. For instance, highly heterogeneous tropical mountain regions, due to their intricate topography, serve as both centers of origin and repositories for species diversity, particularly for range-restricted endemics [4,23]. In contrast, our study revealed that at the fine (5 m) scale, increased microhabitat heterogeneity (as measured by TRI) is associated with reduced species diversity. Based on prior research, we speculate that topographic complexity may affect diversity through three mechanisms. First, at larger scales, complex terrain may promote species turnover and provide refugial or isolating opportunities that facilitate diversity formation [1,4,23]. However, in our study, the overall climatic conditions across the plot were largely homogeneous, meaning that the microclimatic variations and topographic heterogeneity generated at the 5 m scale were insufficient to establish steep climatic gradients or significant isolating effects. On the contrary, such fine-scale habitat fragmentation may impede species dispersal, leading to local community homogenization [5,14]. Second, the patchy distribution of resources may promote ecological niche differentiation, whereby plant species with similar niche requirements become filtered into the same uniform habitat patch. Under fine-scale conditions, homogeneous microhabitats may facilitate niche partitioning, leading to a scenario in which species with comparable requirements are sorted into the same patch. This is reflected in our finding of a negative effect of TRI on NSR. Third, later life-stage heterogeneity—by driving resource patchiness and homogenization—may further enhance niche differentiation, resulting in reduced aggregation of heterospecific individuals in uniform patches. This mechanism is supported by our observation that the negative effect of TRI on the heterospecific neighborhood competition index (HNCI) becomes more pronounced as succession progresses (Figure 4e2), while the negative impact on the conspecific neighborhood competition index (CNCI) gradually reverses towards a positive trend (Figure 4e1). In other words, the enhanced conspecific aggregation in homogeneous patches provides further evidence for this niche-based mechanism. These findings underscore the significance of studying topographic complexity across multiple spatial scales. Although traditional research has documented a positive association between overall topographic complexity and species diversity at larger scales [4], the fine-scale negative relationship observed in our study suggests that the process of habitat homogenization at the neighborhood level may counteract the diversity-promoting effects of large-scale spatial turnover. In other words, as observational scales shift from the neighborhood to the landscape level, the integration of uniformly distributed habitat patches may eventually lead to the complex habitat mosaics observed at broader scales, thereby forming a hierarchical “neighborhood–local–regional–landscape–global” pattern. This integrated perspective emphasizes that forest diversity is maintained not only by environmental filtering driven by climatic gradients [1], but also by fine-scale niche differentiation associated with habitat heterogeneity [2].

The results of this study have important implications for subtropical forest management. For instance, on sun-facing slopes, artificial thinning or selective removal may mitigate strong conspecific competition (CND) and create opportunities for the introduction of drought-tolerant heterospecific species to ease water competition stress. In areas along high TPI zones, which often correspond to ridge-like micro-topographics, prioritizing the conservation of species with deep and shallow root systems could help to maintain resource competition balance. Likewise, in regions with high TRI values, restoration measures such as microtopography modification (e.g., constructing water-retaining gullies) might alleviate habitat fragmentation. However, these correlations require further experimental validation, considering the limitations of our study, such as the spatial scale of observation, the exclusion of belowground processes (fine roots and mycorrhizal networks), and the lack of coupled climate change effects. Looking ahead, future research should integrate multi-scale remote sensing with stable isotope techniques (δ¹⁵N and δ¹³C) to trace resource distribution and develop coupled topography–competition models capable of forecasting forest responses under extreme climate scenarios. Furthermore, controlled experiments using artificial microtopographic modules would be critical for verifying the plasticity of the underlying mechanisms. Ultimately, such an integrative approach could lead to the development of optimized forest management systems that incorporate multidimensional ecological processes, ensuring both the conservation and adaptive management of subtropical forests in a changing global environment.

## 4. Materials and Methods

### 4.1. Study Area and Censuses

The study area is located within the Yaoluoping National Nature Reserve in Yuexi County, Anhui Province, China, situated in the southeastern Dabie Mountains (Figure S2 in the Supplementary Files). This region lies in the transitional zone between the north subtropical and warm temperate zones, bordering Huoshan County of Anhui Province to the north and Yingshan County of Hubei Province to the west [39,40]. The area experiences an annual sunshine duration of 1580–1900 h and an average annual temperature of 12.7 °C. The coldest month (January) has an average temperature of around 2 °C, while the hottest month (July) averages 23 °C. The lowest recorded temperature is −15.2 °C (recorded on 30 January 1977) and the highest is 39.4 °C, with a frost-free period of approximately 220 days. Precipitation is strongly influenced by the monsoon season, with an annual total of about 1400 mm; however, the seasonal distribution is uneven, with summer (June–August) receiving 44.2% of the total, spring (March–May) 31.3%, and winter (December–February) only 8.9%. The region also maintains a high relative humidity of around 80%. Regarding soils, areas below 800–900 m predominantly feature mountainous yellow–brown soil, whereas mountainous brown soil dominates above this elevation; some locales also contain meadow and marsh soils [41]. The zonal vegetation is characterized by a mixed forest of evergreen and deciduous broadleaved trees typical of the north subtropical zone, with a gradual transition to warm temperate deciduous broadleaved forests at higher elevations [37,41].

**Figure 5:**
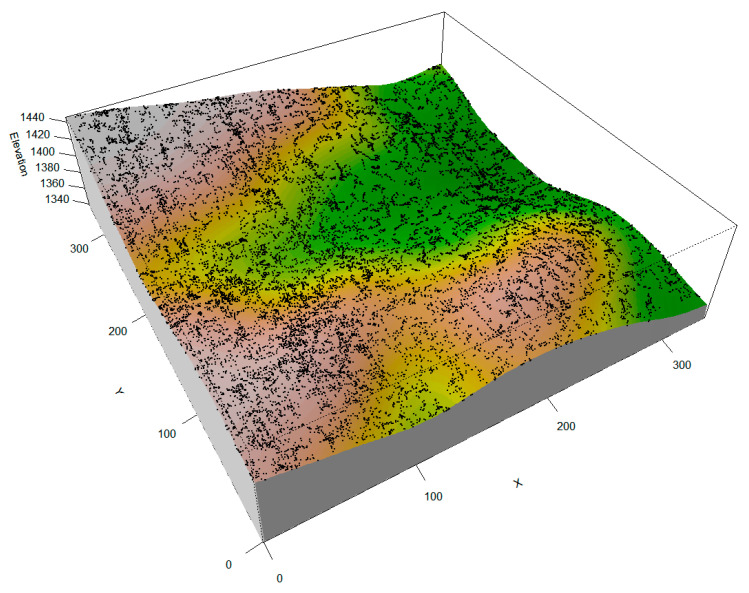
Microtopography of the monitoring plot and tree spatial distribution. This map presents the combined terrain factors, along with the fundamental elevation conditions, with black dots representing the spatial positions of individual trees.

The monitoring plot was established in the southern core area of the nature reserve, where human disturbance is minimal. Covering a total area of 11.56 hectares, the plot is located between 30°57′46.11″–30°57′47.81″ N and 116°04′41.33″–116°04′41.08″ E (Figure 5). The plot was subdivided into 289 quadrats, each measuring 20 m × 20 m, and subsequently subdivided into 4624 working quadrats (each 5 m × 5 m in size). In addition, detailed surveys were conducted within each working quadrat, during which all woody tree species (with a DBH > 1 cm) were mapped, measured for DBH and height, and recorded for species identity and stem count. Within each working quadrat, all arboreal species with a diameter at breast height (DBH) greater than 1 cm were recorded, along with measurements of DBH, tree height, and the number of individuals. A total of 103 tree species were identified, representing 35 families and 57 genera, with the majority being deciduous broadleaved species. Species richness was notably high in families such as Rosaceae, Aceraceae, Ericaceae, and Lauraceae. Additionally, there were 21 co-dominant species with importance values exceeding 0.01, among which *Castanea seguinii*, *Carpinus turczaninowii*, *Cornus kousa*, and *Symplocos paniculata* exhibited the highest occurrence frequencies, each recorded in over 90% of the quadrats (Table S1 in the Supplementary Files). The importance values (IVs) were calculated by combining the percentages of relative abundance, relative dominance (DBH), and relative frequency for these co-dominant species, reflecting their overall ecological significance within the community [37,38].

### 4.2. Microtopographic Factors and Neighborhood Effects

**Figure 6:**
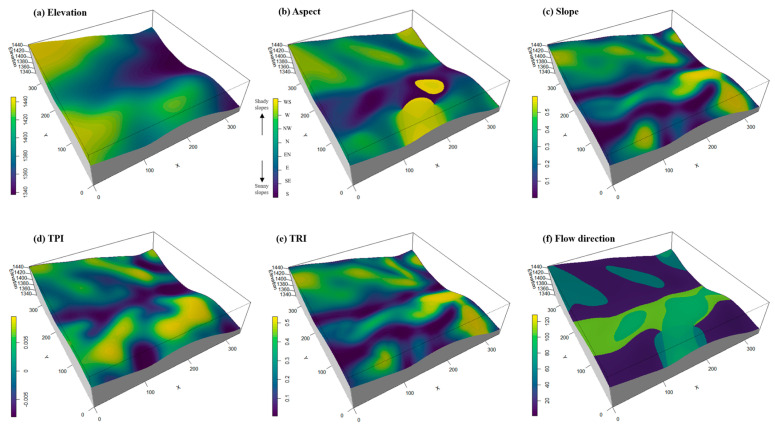
Spatial variation in the microtopographic predictor at the neighborhood scale: elevation (**a**), aspect (**b**), slope (**c**), terrain position index (**d**), terrain ruggedness index (**e**), and flow direction (**f**). The maps were generated using an Epanechnikov kernel with a bandwidth of 5, and the intensity values range from dark blue (low) to green (high).

Within our monitoring plot, key microtopographic parameters—such as mean elevation and slope—were determined for each working quadrat (Figure 6). In accordance with the protocols established by the Center for Tropical Forest Science (CTFS), the entire plot was segmented into 289 quadrats (each 20 m × 20 m), with each quadrat further partitioned into 16 subplots (each 5 m × 5 m). Using a total station, elevation and slope were measured within every subplot (Figure 6a–c), and these data were subsequently used to construct a digital elevation model (DEM) of the study area, from which various microtopographic metrics were extracted [5,42,43]. In this DEM, higher aspect values indicate slopes facing south, whereas lower values denote north-facing, more shaded slopes. To further examine the effects of microtopography on ecological processes in plant communities, additional variables, such as the terrain position index (TPI), terrain ruggedness index (TRI), and flow direction, were computed from the DEM data [5,43,44]. The TPI was calculated by determining the average difference in elevation between each cell and its surrounding cells, thereby assessing terrain position (Figure 6d). The TRI, on the other hand, was quantified as the root mean square of the elevation differences between a central cell and its eight adjacent cells, reflecting the degree of terrain roughness (Figure 6e). The TRI not only quantifies the roughness of the terrain by evaluating the root mean square of elevation differences between adjacent cells, but it also effectively captures the degree of small-scale topographic heterogeneity [43,44].

Flow direction, derived in a similar manner from the DEM, indicates the direction of water movement based on the elevation differences with surrounding cells (Figure 6f); regions with higher flow direction values typically suggest areas prone to water accumulation, potentially offering more water availability to the resident vegetation [43,44]. Finally, microtopographic metrics for focal tree species at various neighborhood scales were computed and visually represented using R Studio (R version 4.2.3), employing the “spatstat” (version 3.0-3) and “raster” (version 3.6-2) packages.

Conspecific neighborhood density (CND) and heterospecific neighborhood density (HND) were quantified by assessing the density of trees belonging to the same species and those belonging to different species, respectively, surrounding each focal tree within predefined radii [10,11,45,46] (refer to Figure S3a,b in the Supplementary Files). In a similar manner, the conspecific neighborhood competition index (CNCI; Figure S3f) and heterospecific neighborhood competition index (HNCI; Figure S3g) were determined by summing the DBH (diameter at breast height) areas of neighboring trees of the same species and different species, respectively [10,11,20,46]. These indices are expressed mathematically as ${NCI}_{i}=\sum_{j\neq i}\frac{\pi D_{j}^{2}}{4}$, where D_j_ denotes the DBH of the neighboring tree j [10,11,12,20,46]. When trees i and j both belong to the same species, the resulting value reflects the intensity of intraspecific resource competition (CNCI); conversely, when trees i and j are of different species, the value indicates the intensity of interspecific resource competition (HNCI). The analyses of CND, HND, CNCI, and HNCI were carried out for radii of 5 m [10,11,12,20,46]. Tree size was evaluated by investigating the relationship between individual tree size, as measured by diameter at breast height (DBH) and tree height (H) [5,45,46]. The DBH and H measurement for each tree were recorded, along with their spatial locations within the plot (Figure S3c,d in the Supplementary Files). 

Neighborhood species richness (NSR; see Figure S3e in the Supplementary Files) serves as a measure of biodiversity by counting the distinct tree species found within a designated radius around each focal tree. For a given focal tree i, the NSR is calculated by summing the number of heterospecific neighbor species, mathematically represented as ${NSR}_{i}=\sum_{j\neq i}N_{j}$, with N representing the number of species recorded for each neighboring tree j [20]. This approach facilitates a detailed investigation into how the immediate species diversity affects the survival of the focal tree. NSR was specifically computed for a radius of 5 m, a distance deemed effective for capturing tree-to-tree interactions [10,11,12,20,46].

### 4.3. Microtopographic Effect on Neighborhood Factor

Linear mixed-effects models (LMMs) were used to build statistical relationships between various microtopographic factors and neighborhood indices of growth, resource competition, and diversity through parameter estimation, analyzing the influence of microtopographic factors and neighborhood effects on tree survival across life history stages. Microtopographic factors (elevation, slope, aspect, TPI, TRI, and flow direction) and neighborhood factors (CND, HND, DBH, H, NSR, CNCI, and HNCI) were included as predictors [5,10,11,20,46]. These aspects include individual growth performance (represented by DBH and tree height, H), local diversity at the neighborhood scale (NSR), spatial density and distribution (CND and HND), as well as resource competition (CNCI and HNCI) [5,47].

We classified trees of all species into distinct life-history stages: sapling1 (DBH < 2.5 cm), sapling2 (2.5 cm ≤ DBH < 5 cm), juvenile1 (5 cm ≤ DBH < 10 cm), juvenile2 (10 cm ≤ DBH < 15 cm), adult1 (15 cm ≤ DBH < 20 cm), and adult2 (DBH ≥ 20 cm). By categorizing individuals in this way, we further quantified the influence of microtopographic factors on tree growth [10,11,12,24,46]. For each life stage of the focal trees, a baseline model was developed to examine how microtopographic characteristics underpin and modulate neighborhood effects, growth conditions, and species interactions. The basic model is expressed as follows:

$${\left(CND/HND/DBH/H/NSR/CNCI/HNCI\right)}_{\;ijp\;}\;\;={{\;\beta }_{1}Elevation\;}_{ijp}+{\;\beta }_{2}{Aspect}_{ijp}+\beta_{3}{Slope}_{ijp}+\beta_{4}{TPI}_{ijp}\;\;+\beta_{5}{TRI}_{ijp}+\beta_{6}{Flow\;direction}_{ijp}\;{+\varepsilon }_{j}\;{+p}_{q}$$

In this equation, the response variable represents one of the measures, i.e., whether it is a growth indicator (DBH or H), a metric of neighborhood diversity (NSR), spatial density/distribution indices (CND, HND), or resource competition (CNCI, HNCI), where *CND/HND/DBH/H/NSR/CNCI/HNCI_ijp_* is the predicted CND/HND/DBH/H/NSR/CNCI/-HNCI of each focal tree i of species j growing in quadrat q. The coefficients β_1_ through β_6_ quantify the directed influence of each microtopographic predictor on these responses. The random effects structure includes *ε_j_* for species-specific baseline differences and variable responses, while *p_q_* captures potential quadrat-level variations. Due to the genetic differences among species with different growth forms, they may exhibit markedly distinct growth patterns and maturity standards. In constructing our models, we treated “species” as a random effect, enabling the model to automatically adjust for each species’ baseline level (random intercept), as well as its sensitivity to microtopographic factors (random slope). This approach effectively absorbs the systematic biases arising from species-specific differences in life-history traits. By estimating a unique baseline and microtopographic response for each species, the inclusion of random effects minimizes the influence of interspecific variability on the overall model, thereby enhancing its robustness and generalizability. Since our study focuses on the neighborhood scale, the minute variations in high-resolution terrain may lead to a strengthening of local couplings and linear relationships in certain ecological processes [48]. For example, the effect of slope might also encompass hydrological influences mediated by TRI. To mitigate this, our design includes (1) a mixed-effects model incorporating quadrat × species random interactions to capture major spatial residual variance and thus reduce the instability in fixed-effect estimates; and (2) a cautious ecological interpretation framework that regards different terrain factors as complementary expressions of terrain complexity, with the collective regulatory effect on ecological processes being the primary focus, rather than the isolated role of each variable. We used the “lme4 1.1-32” package in R to run the basic model for each life stage (based on R version 4.2.3).

## 5. Conclusions

This study demonstrates that microtopography is critical in driving fine-scale heterogeneity, plant interactions, and diversity patterns in subtropical forests. By integrating high-resolution topographic data with neighborhood-scale plant distribution and competition metrics, we reveal that factors such as aspect and TPI play key roles in mediating resource allocation—shaping conspecific clustering, heterospecific competition, and growth trajectories—whereas TRI exerts unexpected effects on local species richness at the 5 m scale.

The interrelationships among microtopographic parameters represent both a technical challenge in data processing and an ecological expression of coupled surface processes. As noted by Dormann et al. (2013), collinearity among different terrain variables derived from DEMs is a common phenomenon [48]. The observed collinearity between slope and TRI in our study conforms to the inherent characteristics of DEM-derived terrain parameters, which is partly due to their similar computational methods. At the neighborhood scale, the narrow computation window used for slope and TRI converges on the same micro-protrusions, thereby synchronously enhancing their responses to terrain discontinuities and resulting in a near-linear association. Notably, this phenomenon diminishes with an increasing testing scale, with significant effects on various neighborhood interactions being detectable at a 10 m resolution. In contrast, other microtopographic factors did not exhibit significant collinearity (VIF < 2), which may be attributed to factors such as (1) elevation, which predominantly governs regional hydrological patterns and is decoupled from local processes; (2) aspect, influenced by heterogeneous solar radiation, whose ecological effects are independent of other terrain parameters; and (3) TPI, reflecting relative elevation differences, but not necessarily linked to absolute slope values. This relative independence among parameters provides a structural basis for analyzing multidimensional terrain effects. Overall, through model optimization and appropriate scale adaptation, we have managed to control the impact of collinearity, thereby elucidating the mechanisms of microtopographic influence on forest neighborhood dynamics.

However, several limitations warrant consideration. First, the absence of direct soil nutrient data, along with the largely unexplored belowground processes (e.g., root networks) and long-term climate interactions, suggest that future research should incorporate soil chemistry, functional traits, isotopic tracing, and experimental manipulations to better resolve species-specific responses to microtopographic gradients. Second, while our uniform DBH-based classification across species may introduce systematic biases, our implementation of mixed-effects models has mitigated genetic and growth form variability; nevertheless, more extensive data should allow for species-group-specific classifications to further enhance model accuracy. Third, despite the mixed-effects approach effectively reducing interspecific and spatial heterogeneity, the inherent collinearity among DEM-derived predictors—particularly between slope and TRI—limits the independent evaluation of these factors. Future studies employing dimension reduction techniques or higher-resolution topographic data could help disentangle these effects and further refine our understanding of microtopographic effects on forest dynamics. Together, these findings underscore the vital role of microtopography in creating niche opportunities that balance competitive exclusion and coexistence, and they provide a framework for predicting forest resilience and informing restoration strategies in the face of global environmental change.

## Data Availability

Data are contained within the article and supplementary materials.

## Supplementary Materials

The following Supplementary Files can be downloaded at: https://www.mdpi.com/article/10.3390/plants14060870/s1, Figure S1. Parameter estimates for the effects of microtopographic factors on seven neighborhood effect metrics at a scale of 2.5 m (a–f) and 10 m (a1–f1). Figure S2. Schematic diagram of the monitoring plot. Figure S3. Spatial variation of seven neighborhood effect metrics at a scale of 5 m. (a) CND, (b) HND, (c) DBH, (d) H, (e) NSR, (f) CNCI, and (g) CNCI. The maps were generated using an Epanechnikov kernel with a bandwidth of 5, and the intensity values range from green (low) to yellow (high). Table S1. Basic characteristics of the 21 co-dominant tree species (IV > 0.01) in a dynamic forest plot in Yaoluoping, Anhui Province, China.
