# Supplementary material for: Effects of Microtopography on Neighborhood Diversity and Competition in Subtropical Forests
Source: Plants (Basel). 2025 Mar 11;14(6):870. doi: 10.3390/plants14060870 (PMC11945650; doi:10.3390/plants14060870)
Supplement: Supplementary file 1 [file plants-14-00870-s001.zip › plants-3506899-supplementary.pdf]

Supplementary Files

Figure S1. Parameter estimates for the effects of microtopographic on seven neighborhood effect metrics at a scale of 2.5 m (a, b, c, d, e, f) and 10 m (a1, b1, c1, d1, e1, f1).

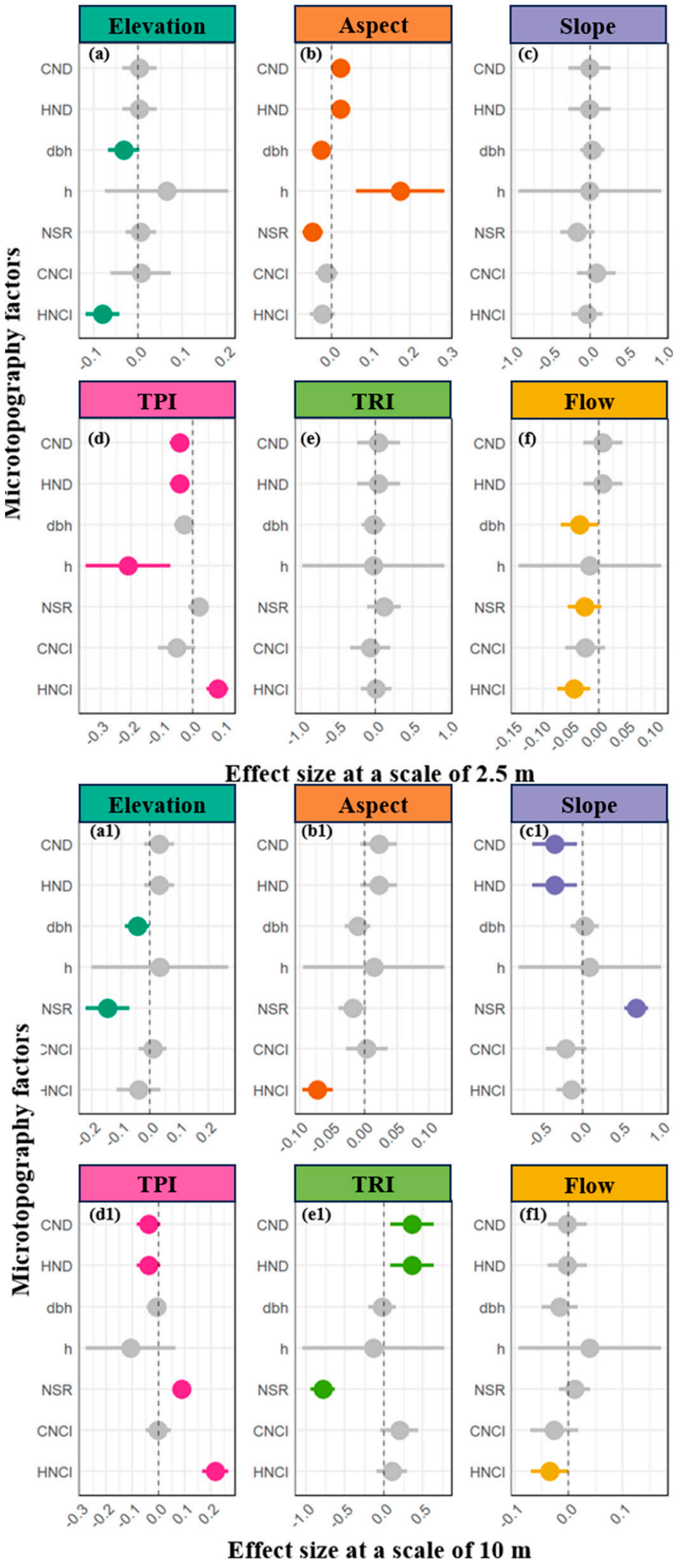

Figure S2. Schematic diagram of the monitoring plot

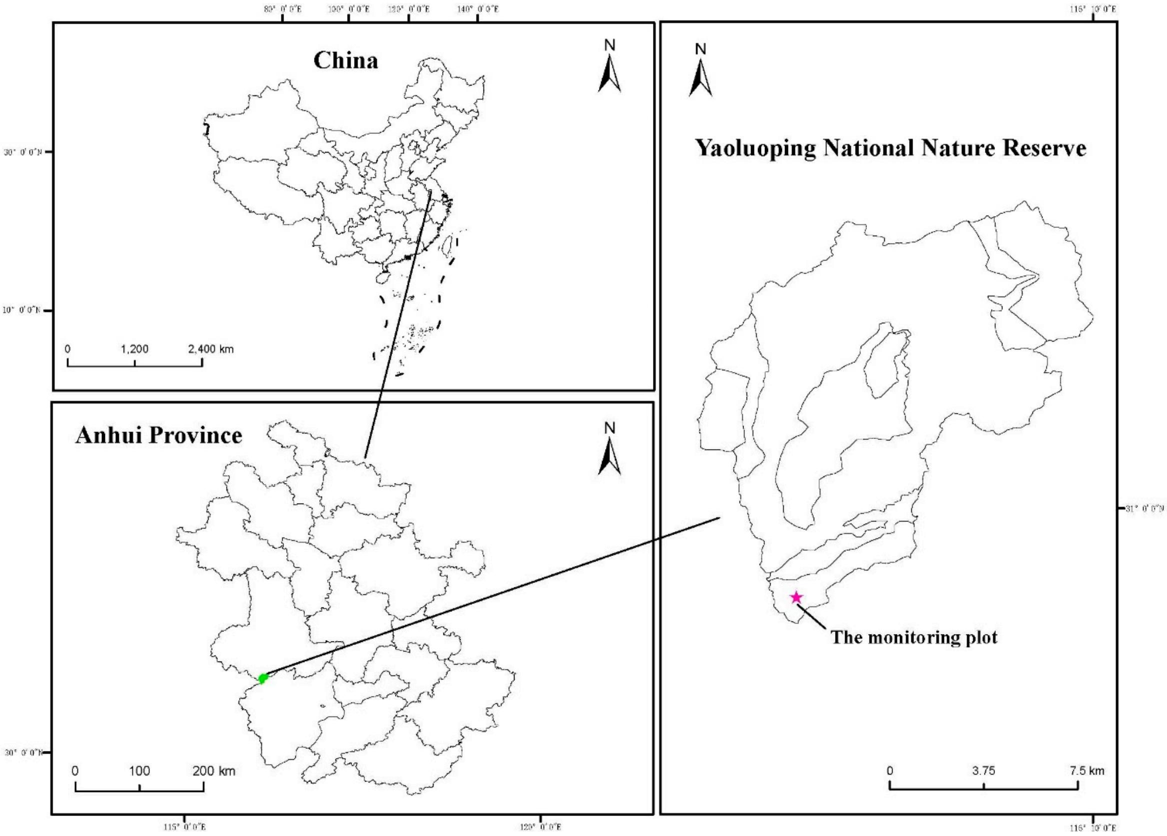

Figure S3. Spatial variation of seven neighborhood effect metrics at a scale of 5 m. (a)CND, (b)HND, (c)DBH, (d)H, (e)NSR, (f)CNCI, (g)CNCI. The maps were generated using an Epanechnikov kernel with a bandwidth of 5, and the intensity values range from green (low) to yellow (high).

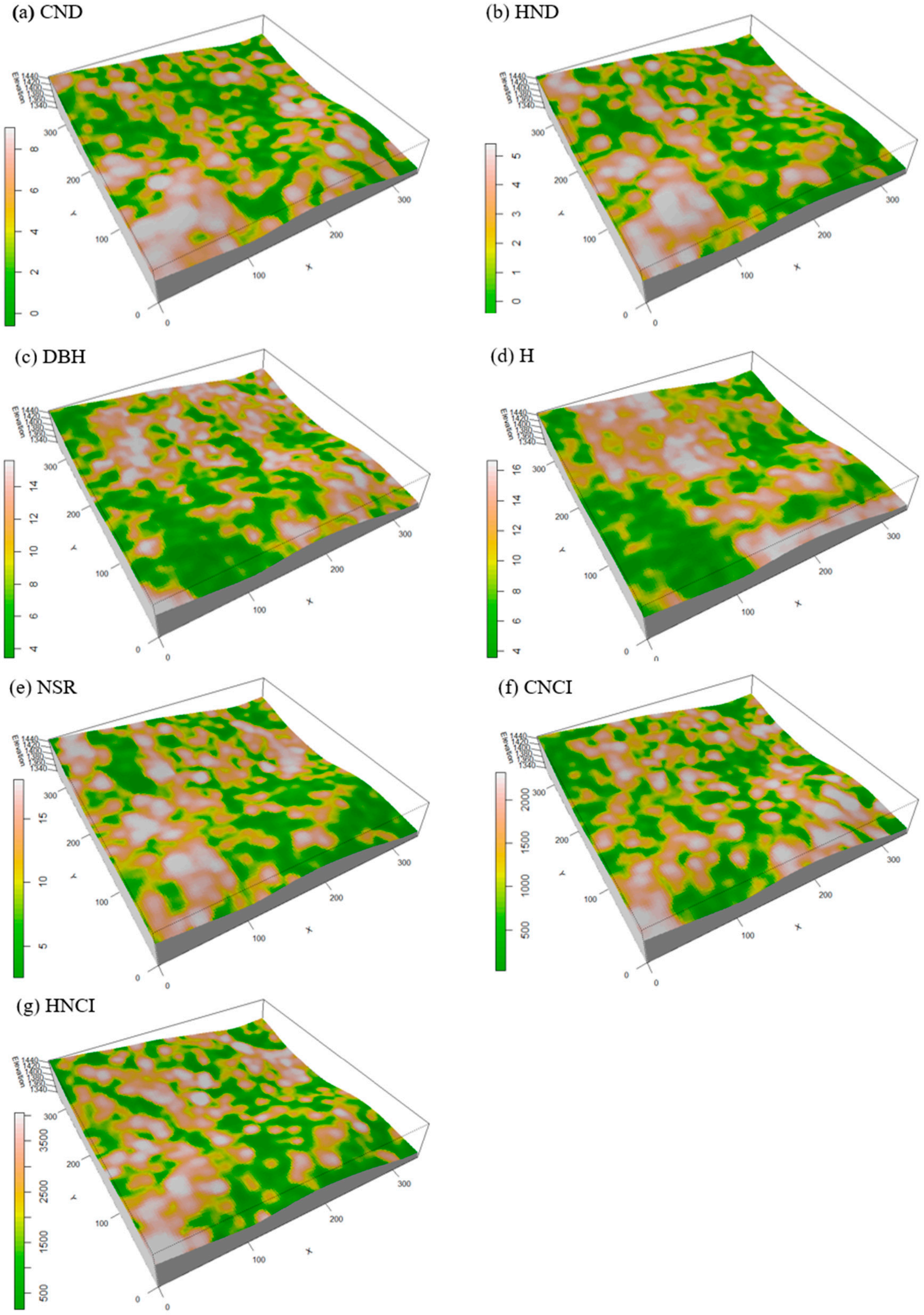

Table S1. Basic characteristics of the 21 co-dominant tree species (IV > 0.01) in dynamic forest plot in the Yaoluoping, Anhui province, China.

Table S1 Importance value of the 21 co-dominant species (IV＞0.01)

Code	Species	Importance Value (%)	Leaf habits	Mean DBH (cm)
1	*Castanea seguinii* Dode	0.214	Deciduous	14.47
2	*Carpinus turczaninowii* Hance	0.093	Deciduous	8.45
3	*Cornus kousa subsp. chinensis* (Osborn) Q. Y. Xiang	0.070	Deciduous	6.30
4	*Symplocos paniculata* (Thunb.) Miq.	0.061	Deciduous	4.53
5	*Pinus taiwanensis* Hayata	0.052	Evergreen	15.17
6	*Sorbus alnifolia* (Sieb. et Zucc.) K. Koch	0.050	Deciduous	8.97
7	*Corylopsis sinensis* Hemsl.	0.047	Deciduous	4.59
8	*Cornus controversa* Hemsley	0.040	Deciduous	11.65
9	*Pterostyrax corymbosus* Sieb. et Zucc.	0.034	Deciduous	16.18
10	*Rhododendron simsii* Planch.	0.028	Evergreen	2.74
11	*Lindera obtusiloba* Bl. Mus. Bot.	0.025	Deciduous	4.98
12	*Acer nikoense* Maxim.	0.023	Deciduous	5.97
13	*Cerasus serrulata* (Lindl.) G. Don ex London	0.017	Deciduous	9.90
14	*Photinia beauverdiana* C. K. Schneid.	0.016	Deciduous	5.49
15	*Styrax obassia* Sieb. Et Zucc.	0.015	Deciduous	8.65
16	*Weigela japonica*	0.015	Deciduous	5.48
17	*Quercus serrata*	0.012	Deciduous	9.25
18	*Quercus aliena*	0.012	Deciduous	12.15
19	*Meliosma flexuosa*	0.012	Deciduous	3.49
20	*Toxicodendron succedaneum*	0.011	Deciduous	9.29
21	*Acer pictum subsp. mono* (Maximowicz) H. Ohashi	0.011	Deciduous	7.14
